# 8-Isoprostane in chronic periodontitis and type II diabetes: Exploring the link

**DOI:** 10.15171/joddd.2018.039

**Published:** 2018-12-19

**Authors:** Arati C Koregol, Nagaraj B Kalburgi, Sireesha kanniappa sadasivan, shivaraj warad, Apoorva kamat wagh, Tivin Thomas, Priya sinha

**Affiliations:** ^1^Department of Periodontics, P.M. Nadagouda Memorial Dental College and Hospital, Bagalkot, India

**Keywords:** Antioxidant, chronic periodontitis, diabetes mellitus, isoprostanes, reactive oxygen species, saliva

## Abstract

***Background.*** Reactive oxygen species (ROS) are associated with the pathogenesis of inflammatory diseases and have a direct or indirect role in tissue damage constituting oxidative stress. ROS are also involved in impairment of β-cell function during development of diabetes, which leads to genetic ablation of KATP channels, triggering up-regulation of antioxidant enzymes. Several markers of lipid peroxidation, protein oxidation and DNA damage induced by ROS can be measured. Over the last decade, isoprostanes have been considered as the best markers of lipid peroxidation. The aim of this study was to determine the presence of 8-isoprostane in healthy, chronic periodontitis and chronic periodontitis subjects with type II diabetes and to find the correlation between 8-isoprostane levels among groups and with clinical parameters like gingival index, probing depth and clinical attachment levels.

***Methods.*** Ninety subjects were selected and divided into 3 groups: healthy, chronic periodontitis and chronic periodontitis subjects with type II diabetes (n=30 each). Saliva was collected from these subjects after obtaining consent and analyzed for 8-isoprostane levels using ELISA kit. Statistical analysis was performed using Kruskal-Wallis test, Mann-Whitney U test and Spearman’s correlation coefficient (P<0.001).

***Results.*** Statistically significant difference was found in the levels of 8-isoprostane between healthy, chronic periodontitis and chronic periodontitis subjects with type II diabetes and with all clinical parameters.

***Conclusion.*** 8-isoprostane can be considered as a pathophysiological marker to measure oxidative stress in periodontal diseases.

## Introduction

Oxidative stress (OS) occurs due to an imbalance between pro-oxidant/antioxidant status, resulting in the generation of reactive oxygen species (ROS) and subsequent modification of biomolecules such as proteins, lipids and nucleic acids and changes in the organism’s structure and functions.^1^ Oxidative stress also has a beneficial role in the physiologic adaptation and in the regulation of intracellular signal transduction. Tissue damage ensues due to a number of enzyme- and non-enzyme-mediated biochemical reactions which tremendously produce reactive intermediate compounds called “free radicals”.^2^ Free radicals have been defined as “Any species capable of independent existence that contain one or more unpaired electrons”.^3^

Excess generation of ROS is seen in inflammatory conditions in the oral cavity like periodontitis. Periodontitis is a multifactorial disease due to the interplay between various microbiologic, genetic, immunologic, environmental and behavioral risk factors that are responsible for the onset, course and severity of disease. Various studies have emphasized the association between periodontal disease and oxidative stress in recent years.^4,5^ There are several mechanisms involved in the genesis of oxidative stress in diabetic patients.

Diabetes mellitus type II is characterized by hyperglycemia, promoting microvascular damage through four mechanisms (Acc to Brownlee):^6^

(i) increased flux of glucose through the polyol pathway
(ii) intracellular production of advanced glycation end-products
(iii) persistent protein kinase C activation
(iv) increased hexosamine pathway activity

There is a direct relationship between the circulating blood glucose levels and glucose variability. The sixth complication of diabetes is periodontitis. Diabetes and periodontitis ought to have bidirectional relationship. Lipid peroxidation (LPO) is the prominent marker of oxidative stress.,^7,8^

Oxidative damage of cellular membranes has been considered as a common mechanism in various biopathological conditions. Oxidative damage can be measured by either primary or secondary end-products of lipid peroxidation. Primary end-products of lipid peroxidation include conjugated dienes and lipid hydroperoxides, while secondary end-products include thiobarbituric reactive substances (TBARS), gaseous alkanes and F2- isoprostanes (F2-IsoPs).^9^ Due to its specificity, reliability and its detection in various biological fluids, F2-isoprostanes are considered reliable biomarkers of lipid peroxidation over the last decade and could therefore be used as potential indicators of oxidative stress in diverse conditions.^10^

Isoprostanes (IsoP) were first discovered in 1990 as a series of prostaglandin(PG)-like compounds generated in vivo by the free radical-catalyzed peroxidation of arachidonic acid and in vitro by nonenzymatic peroxidation of purified PUFA.^11^ IsoPs might be formed by either of two routes of peroxidation: an endoperoxide mechanism or a dioxetane mechanism.,^10^ F2-IsoPs are stable molecules and are detectable in all human tissues and biological fluids, including plasma, urine, bronchoalveolar lavage fluid, cerebrospinal fluid and bile.^12^

With this in mind, the present study was designed on 8-isoprostane (8-IsoP) to establish the link between chronic periodontitis and type II diabetes. 8-IsoP levels were estimated in healthy, chronic periodontitis and chronic periodontitis with type II diabetes and further compared and correlated with the clinical parameters. To the best of our knowledge this is among the footmark studies to qualify 8-IsoP as a biomarker in chronic periodontitis subjects with and without type II diabetes.

## Methods

### Study population

A total of 90 samples aged 18−60 years were selected from the outpatient section of the Department of Periodontics, P.M. Nadagouda Memorial Dental College, Bagalkot, Karnataka, India. The protocol for this cross-sectional study was approved by the Institutional Ethics Committee. Prior to enrolment in the study, an informed consent was obtained from the candidates who fulfilled the inclusion criteria.

After clinical examination, the subjects were divided into three groups of 30 each. Group I consisted of 30 healthy subjects showing absence of clinical and radiographic manifestations of periodontal disease, with at least 20 teeth present. Group II comprised of 30 subjects diagnosed with chronic periodontitis with the presence of bleeding on probing and clinical attachment level of 3 mm or more in more than 30% of the teeth in the mouth.^13^ Group III consisted of 30 subjects fulfilling the criteria of Group II in addition to having type II diabetes (Diagnosis of type 2 diabetes mellitus was made according to American Diabetes Association standards in which individuals with fasting plasma glucose (FPG) ≥126 mg/dL (7.0 mmol/L) or symptoms (such as polyuria, polydipsia, unexplained weight loss) and a random plasma glucose ≥200 mg/dL (11.1 mmol/L) or plasma glucose ≥200 mg/dL (11.1 mmol/L) 2 hours after a 75-g glucose load or HbA1C ≥6.5%.).^14^

Subjects suffering from systemic conditions (rheumatic fever, heart diseases, hypertension, liver and kidney disease), any infection requiring prophylactic antibiotic therapy, pregnant females, lactating women, subjects on hormonal contraceptives or on hormone replacement therapy, on steroids and NSAIDs (for previous three months) or on vitamin supplements, smokers and tobacco chewers, alcoholics and having undergone scaling and root planing in the past six months were excluded from the study as they proved to affect the levels of 8-isoprostane.^23,24^

### Determination of clinical parameters

After proper grouping of the subjects, a full-mouth periodontal examination was performed by a single examiner. The periodontal parameters like pocket depth (PD), clinical attachment level (CAL) and gingival index (GI) (Loe and Silness 1963) were assessed using a Williams periodontal probe by a single examiner.

### Sample collection and processing

Unstimulated whole saliva samples were collected as it represents major intraoral conditions regarding saliva status and composition.^15^ Unstimulated whole saliva samples were collected on the same day. The subjects were instructed to refrain from food and beverages except water, perform oral hygiene measures or chew or smoke anything for two hours before saliva collection. To minimize circadian influences, all the salivary samples were collected from 9 a.m. to 12 p.m. Stimulation of saliva was prevented during sample collection.

During collection, the subjects were comfortably seated with eyes open, head tilted slightly forward and making minimal orofacial movements, and saliva was allowed to accumulate in the floor of the mouth. The subjects were instructed not to speak or swallow during collection. The subjects were then told to spit into the 2-mL Eppendorf tube every 60 seconds for 10 minutes or when the subject experienced an urge to swallow the fluid accumulating in the floor of the mouth. Around 2 mL of whole saliva was collected. The procedures were assisted by the same person.

The salivary samples collected from the selected subjects were centrifuged at 1000 ×g for 20 minutes at 4°C immediately after collection to remove cells and debris. The supernatants were divided and transferred to graduated test tubes, frozen and kept at -80°C until analyzed. Furthermore, the salivary samples were thawed and analyzed by ELISA (ELAB SCIENCE CO. LTD).

### Statistical Analysis

Results on continuous measurements are presented as mean ± SD. Results on categorical measurements are presented in number (%). Differences were considered to be statistically significant at P<0.05. Kruskal-Wallis test was used to compare the three groups with respect to GI, PPD and CAL. Mann-Whitney U test was used for pair-wise comparisons between any two groups. Spearman’s rank correlation was used to find out the correlation between 8IsoP levels and GI, PPD and CAL in the three groups. SPSS 21.0 was used for the analysis of the data. Microsoft Word and Excel were used to generate graphs and tables.

## Results

### Participant Demographics

According to demographic data of 90 study subjects, the mean age of the study subjects was 41.55±7.12 years and gender distribution was 39 males (35.1%) and 51 females (45.9%). These data are presented in Tables 1 and 2, respectively.

### Comparison of 8IsoP levels in all the groups

The mean age of diabetes group was found to be the highest (Table 1). All the samples in each group tested positive for 8-IsoP assay. The mean 8-IsoP concentration in saliva was the highest in healthy (1092.52±310.01 pg/mL) (P<0.001), followed by chronic periodontitis (564.94±354.80 pg/mL) (P<0.001), wuth the least in chronic periodontitis with type II diabetes (485.39±288.49 pg/mL) (P<0.001) as shown in Table 1.

**Table 1:** Comparison of the study parameters between the healthy and chronic periodontitis subjects

**Group**		**N**	**Mean (SD)**	**Kruskal-Wallis test**	**Mann-Whitney U test (P-value)**			
				**Chi-squared value**	**P-value**	**1 vs 2**	**1 vs 3**	**2 vs 3**
**AGE**	**Healthy**	30	28.30 (5.08)	56.955	<0.001*	<0.001*	<0.001*	0.04*
	**CP**	30	45.93 (8.30)					
	**CP with diabetes**	30	50.43 (8.00)					
**Count (pg/mL)**	**Healthy**	30	1092.52 (310.01)	35.497	<0.001*	<0.001*	<0.001*	0.39(NS)
	**CP**	30	564.94 (354.80)					
	**CP with diabetes**	30	485.39 (288.49)					

*P<0.05 statistically significant P>0.05 Non-significant, NS

### Correlation of 8 IsoP levels in all the groups

A statistically significant relation was found when salivary 8IsoP levels of healthy and chronic periodontitis groups were compared and when healthy and chronic periodontitis with type II diabetes groups were compared. The differences in the mean salivary 8-IsoP concentrations were statistically non-significant when chronic periodontitis and chronic periodontitis with type II diabetes groups were compared.

### Correlation of 8 IsoP levels with the clinical parameters

A significant positive correlation was found between 8-IsoP levels and the clinical parameters like GI, PD and CAL as depicted in Table 3. However, 8-IsoP levels did not have significant correlation with age and gender (Table 2).

**Table 2:** Comparison of counts between genders in each study group

**Group**	**SEX**	**N**	**Mean (SD**	**Mann-Whitney U test**	
				**U statistic**	**P-value**
**Healthy**	Male	14	1171.00 (338.96)	72.00	0.10(NS)
	Female	16	1023.86 (274.68)		
**CP**	Male	11	569.26 (404.74)	103.00	0.95(NS)
	Female	19	562.44 (334.35)		
**CP with diabetes**	Male	14	539.13 (244.49)	76.00	0.14(NS)
	Female	16	438.37 (322.56)		

Mann-Whitney U test *P<0.05 statistically significant P>0.05 Non-significant, NS

**Table 3:** Correlation between study parameters in healthy and chronic periodontitis subjects

		**Count (pg/mL)**		
		**Healthy**	**CP**	**CP with diabetes**
**GI**	Correlation coefficient	0.89	0.99	0.99
	P-value	<0.001*	<0.001*	<0.001*
**PPD**	Correlation coefficient	0.83	0.84	0.70
	P-value	<0.001*	<0.001*	<0.001*
**CAL**	Correlation coefficient	.	0.93	0.89
	p-value	.	<0.001*	<0.001*

Spearman’s correlation test *P<0.05 statistically significant P>0.05 Non-significant, NS

## Discussion

The bulk of periodontal tissue destruction is caused by an inappropriate host response to microorganisms and their products. More specifically, a loss of homeostatic balance between proteolytic enzymes, their inhibitors, reactive oxygen species (ROS); the antioxidant defense systems that protect and repair vital tissues, cells and molecular components are responsible for periodontal tissue destruction. Some bacterial end products lead to priming of PMNs. A minimum oxygen tension of about 1% and a pH of 7.0−7.5 are required for significant generation of ROS by neutrophils. Both these conditions are found within periodontal pockets, indicating that excess ROS production is a significant factor for periodontal tissue damage. Periodontal inflammation like diabetes also leads to the accumulation of advanced glycation end-products in periodontal and gingival tissues, thus signifying an interaction between diabetes and periodontitis.^16,17^ These intracellular glycations of the mitochondrial respiratory chain proteins have been found to produce more reactive oxygen species, which further promote the formation of advanced glycation end-products. This leads to a situation in which advanced glycation end-products increase in periodontitis and diabetes, the process being called ‘metabolic memory’.^18^

The subgingival plaque is the main etiologic agent for the initiation of inflammatory changes in the periodontal tissue. Lipopolysaccharides (LPS) and DNA from these bacteria cause the stimulation of both activating protein-1 and nuclear factor-kb[beta] pathways in the gingival fibroblast via cluster differentiation (CD14) and toll-like receptors (TLR-4), further causing production of inflammatory cytokines. Activation of NF-kb(beta) and AP-1 causes activation of osteoclasts and increases the concentration of MMP (matrix metalloproteinases), which ultimately results in tissue damage by over-production of lipid peroxides, inflammatory mediators and oxidized proteins.^19^ 8-IsoP is one of the lipid peroxides with an important role in oxidative damage by undergoing chain breakage and membrane permeability, leading to apoptosis which is the programmed cell death.

Saliva is a biofluid that contains components derived from the mucosal surfaces, gingival crevices, and tooth surfaces of the mouth. Saliva also contains microorganisms that colonize the oral cavity and other exogenous substances; therefore, it can possibly provide an insight into the relationship of the host and the environment.^20^ Saliva is non-invasive and contains enzymes, proteins, hormones and other biomarkers that can be used as indicators of various diseases. It has advantages over other biological fluids like GCF, serum and plasma and does not require the help of trained personnel and is readily available. Saliva might reflect the physiological condition of the body and therefore is often called “the mirror of the health of the organism”.

In the present research healthy subjects showed higher levels of 8-IsoP as compared to chronic periodontitis subjects, which was statistically significant (Table 1). This study is consistent with a study by Ide et al,^21^ who reported that under healthy conditions, cellular respiration is the dominant source of ROS in the mitochondria. Greater oxidative stress in men is due to increased production of ROS and/or reduced activity of antioxidants. Therefore, a higher baseline metabolic rate in men than in women might have contributed to a higher level of oxidative stress in men in the present study. Furthermore, the antioxidant properties of estrogen might contribute to decreased oxidative stress. We hypothesize that this could be a probable reason for the increased 8-IsoP levels in healthy subjects.

In the present research, 8-IsoP levels are increased in chronic periodontitis compared to chronic periodontitis with type II diabetes, which was statistically significant (Table 1). The results are concurrent with the study by Singh et al^22^ in 2012, who reported that in diabetes, beta cells are tremendously sensitive to oxidative stress. In beta cells, important targets of an oxidant insult are cell metabolism and K_ATP_ channels. The oxidant-induced alterations of K_ATP_ channel activity lead to oxidant-induced dysfunction because genetic ablation of K_ATP_ channels attenuates the effects of oxidative stress on beta cell function. In addition to the effects on metabolism, the interference of oxidants with mitochondria plays a key role in apoptosis. Loss of functional K_ATP_ channels leads to the upregulation of antioxidant enzymes. This process depends on cytosolic Ca^2+^. We hypothesize that this could be the reason for increased 8-IsoP levels in chronic periodontitis subjects. The present results were contradictory to the findings reported by Su et al,^23^ in which significantly higher levels of 8-epi PGF2α were found in saliva of 58 chronic periodontitis subjects and in another study by Pradeep et al,^24^ where gingival crevicular fluid 8-IsoP levels of 26 chronic periodontitis subjects were higher.

A statistically significant negative correlation was found between 8-IsoP levels and clinical parameters like probing depth, clinical attachment levels and gingival index among healthy, chronic periodontitis, chronic periodontitis with type II diabetes group (Table 3). The reason for this could be the fact that matrix metalloproteins play an important role in periodontal tissue destruction.^25^ 8-IsoP inhibit these MMPs like MMP-2 and MMP-9, which suggests its protective role in preventing disease progression in periodontitis.^25^

## Conclusion

It is believed that oxidant stress plays an important role in the pathophysiology of numerous human diseases. That is why it is very important to develop methods that could accurately assess oxidative injury in vivo. There is a need for further research on isoprostanes and to increase knowledge about their metabolites which have been suggested to be more accurate indexes of systemic oxidant stress status. The present study focused on saliva as it is a simple, reliable and inexpensive chairside diagnostic tool when the results are contradictory when compared with other tissue fluids like GCF, plasma, serum etc. Therfore 8-isoprostane can be considered as the pathophysiological marker to determine the oxidative stress. For establishing a link, further studies are necessary with larger sample sizes to detect isoprostane levels in saliva through highly sensitive procedures like mass spectrometry, high-performance liquid chromatography (HPLC) and gas chromatography in subjects with chronic periodontitis and type II diabetes.

## Authors’ contributions

Please add authors’ contributions according to the Journal format.

## Acknowledgments

Authors deny any conflict of interest.

## Funding

Please add funding statement.

## Competing interests

Please add a statement for competing interests.

## Ethics approval

Please add ethics clearance statement or code.
